# Parental Education Key to Health for Parents and Children

**Published:** 2008-03-15

**Authors:** Joanne Appleton-Arnaud

**Affiliations:** Boston Adult Literacy Fund, Boston, Massachusetts

## To the Editor:

Although the goal of reducing school dropout rates is laudable and would surely produce many of the benefits suggested by Freudenberg and Ruglis (1), the simple truth is that this approach will not produce the most benefit for the effort and funding required. There are two reasons for this. First, the history of public education policy is littered with failed attempts to tackle the problem of high-school dropouts in a variety of communities. Second, there is a surer route to improving health by increasing literacy: educate parents. This strategy will also increase the likelihood of children staying in school.

As long-time advocates of adult education, we know about the close relationship between educational attainment and health. U.S. death rates overall and for specific diseases such as cancer and heart disease are higher (in some cases more than double) for adults with less than 12 years of education than they are for people with more than 12 years of education (2). Low literacy levels add an estimated $73 billion to health care costs per year (3).

Just as important, however, are the effects that the educational level of parents have on their children's level of education. Children flourish when their parents have basic literacy skills, functional English, and high school credentials (4,5). Moreover, children whose parents have not completed high school and who are unemployed are five times more likely to drop out of school than are the children of parents who have completed high school and who are gainfully employed (6). The factor that most strongly correlates with the educational achievement of children is the educational achievement of their parents (7). Research findings strongly suggest that attempting to stem dropout rates at the point of exit is a mistaken strategy. Instead, efforts should be directed at ensuring that parents are equipped to inspire, encourage, support, and nurture the educational efforts of their children.

To produce the benefits suggested by Freudenberg and Ruglis, a strong investment in adult basic education (General Educational Development [GED] programs, adult literacy classes, and English-for-speakers-of-other-languages [ESOL] programs) would produce benefits for adults and children: they would assist adults in improving their own lives and health, which would lead to a home environment for children in which they would have the greatest opportunity to succeed.

**Read the author's reply**
